# Supplementary material for: Genetic Testing and Surveillance of Young Breast Cancer Survivors and Blood Relatives: A Cluster Randomized Trial
Source: Cancers (Basel). 2020 Sep 5;12(9):2526. doi: 10.3390/cancers12092526 (PMC7563571; doi:10.3390/cancers12092526)
Supplement: Supplementary file 1 [file cancers-12-02526-s001.pdf]

Breast Cancer and Your Family: How to Improve Screening and Monitoring

A guide for young breast cancer survivors and their female relatives

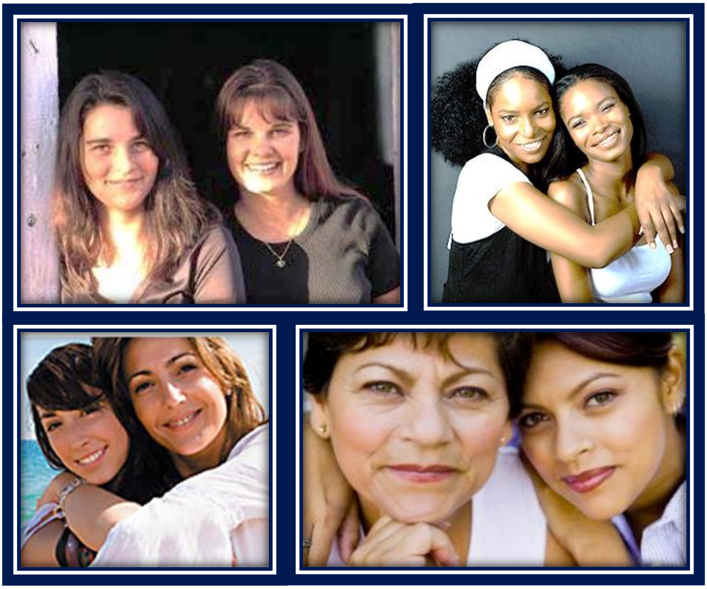

This booklet was created by
the ***University of Michigan School of Nursing***
in collaboration with
the ***Michigan Department of Community Health***
with funding from
the ***Centers for Disease Control and Prevention***

University of Michigan SCHOOL OF NURSING

Michigan Department of Community Health
MDCH
Rick Snyder, Governor
James K. Haveman, Director

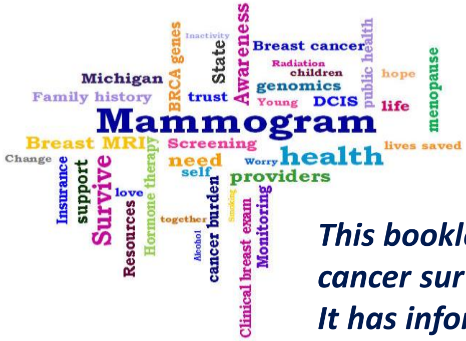

This booklet was created for young breast cancer survivors and their family members. It has information about breast cancer screenings and tips about low cost screening that you may need to know.

Basics About Breast Cancer

Breast cancer is the most common cancer in women. Most breast cancer happens by chance and all women are at risk of getting it. But some women are in a higher-risk group. A health care provider can talk to you more about breast cancer risk factors and breast cancer screenings.

Some Women Are in a Higher-Risk Group

Breast cancer can run in families. This may be due to shared genes, environment and behaviors. When breast cancer runs in a family, some members are at higher risk. Also, the chances of getting it at a younger age are greater.

If you had breast cancer at a young age, your chances of getting another cancer may be higher than other women who got breast cancer later in life. It is important that you watch for early signs of cancer. Frequent screenings help your health care provider pay close attention to your health.

If someone in your family had breast cancer at a young age, you are in this high-risk group. This does not mean you will get breast cancer for sure. It means that your chances may be higher than other women your age. It is important that you start screenings at a younger age than women without a strong family history.

Genetic Services For Women at Higher Risk

What Is Genetic Counseling?

Genetic counseling is suggested for women in a higher-risk group. Genetic services are provided by a health care provider who has special training. Genetic specialists suggest tests that look for changes in genes that are passed down in your family. Several genes are connected to breast cancer, such as *BRCA1, BRCA2, Chek2, PTEN, p53, STK11,* and *CDH1.* **If you had breast cancer before**, this helps find out your chances for getting it another time. **If you never had breast cancer**, this helps find out your chances for getting it. Cancer genetic specialists can also suggest ways to lower this risk. **If you choose to have genetic testing**, genetic specialists can explain your results. They can also suggest ways to lower your cancer risk.

What Happens In Genetic Counseling?

A genetic specialist asks questions about your health and your family's health history. They review your chances of having changes in specific genes that are connected to breast cancer. You will get information about genetic testing, screening, and prevention options. They can also help you decide if genetic testing is the best choice for you and your family.

What do Genetic Services Cost?

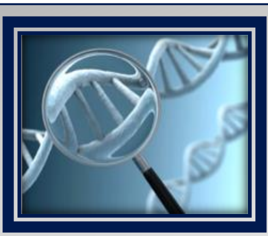

The visit to a specialist costs the same as any other medical specialty visit. Genetic testing is not covered by all insurance plans. Genetic specialists will talk to your insurance and to financial aid programs of the testing companies to find out your cost **before the test is ordered**. They also explore other co-pay programs to help you with the cost of testing.

For a cancer genetics provider near you, see the list on page 7.
For more information on cancer genetics visit www.migrc.org, www.michigancancer.org, or www.nccn.org; call 1-866-852-1247; or email genetics@michigan.gov.

Breast Cancer Screening For Higher-Risk Women

Women in a higher-risk group need to have frequent screenings. These screenings are

- **mammogram** once a year
- **breast exam by a health care provider** once or twice a year
- maybe a **breast MRI**

If you had breast cancer before, these tests will help your health care provider watch for signs of another breast cancer.

If you never had breast cancer, you may need to start screenings at a younger age. Discuss with your health care provider what the best age is for you to begin screening.

Please read these two pages if you never had a mammogram.
See page 6 for tips for low cost screening.

What is a mammogram and why should I get one?

Mammograms are x-ray pictures of your breasts. They are the best way to find breast cancer early, before a lump can be felt. Finding cancer early, when it is most treatable, can increase your chances for long-term survival. It is important to have mammograms regularly, even if you have had normal ones in the past. It helps your provider track changes in your breasts over time and look for early signs of cancer.

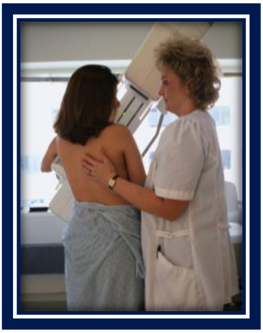

Are mammograms painful?

During the mammogram, a machine presses each breast for a few moments so a good picture can be taken. Most women find that having a mammogram is a little uncomfortable. But, many women say it is worth it for the peace of mind.

What happens if my mammogram is abnormal?

If your mammogram is abnormal or more tests are needed, do not panic. This does not always mean you have cancer. Many women need follow-up tests. You may see specialists who are experts in finding breast problems.

Am I too young to get a mammogram?

No. Because of your family history you are in a **higher-risk group**. This means you have higher chances of getting breast cancer than other women your age. You may need to start getting mammograms at an **earlier age.**

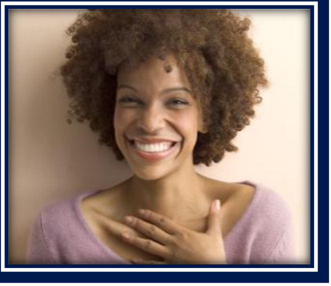

What if my doctor never mentioned it?

Talk to your provider about **your family history and your risk for breast cancer**. Ask for a referral to get a mammogram. Or, you can contact a nearby facility directly.

"My sister urged me to go for a mammogram, and we found my breast cancer when it was very small. Ten years later, I'm here to enjoy my family."

What if I am pregnant, nursing, or getting fertility treatments?

Mammograms are done during pregnancy only if you notice a lump or a change in your breast. Mammograms are safe while breastfeeding. If you are getting fertility treatments, talk with your provider about screenings for you.

Other Screenings

Clinical Breast Exams (CBE)

Clinical breast exams are additional tools to find breast cancer. They may be part of your regular checkups. Your health care provider looks for lumps or other problems in the breasts and underarms. Clinical breast exams cannot replace mammograms. A mammogram may find a lump that is too small to feel.

Breast Magnetic Resonance Images (MRI)

MRIs **may be used in addition** to mammograms and clinical breast exams. This is another way of taking pictures of your breasts. During an MRI, you have an IV and you lie on your stomach on a special bed. MRIs are **NOT for ALL women in a higher risk group**. Ask your provider if breast MRIs, in addition to mammograms, is the right screening for you.

Breast Awareness

Breast awareness may help women know their breasts and look for changes. Check your breasts at the end of your periods. At that time, breasts may be less tender and may be easier to check for lumps.

Scheduling Your Breast Screening

Call your provider for a referral or make an appointment yourself. If you have health insurance, call the customer service number on the back of your card to find out about coverage.

To locate a mammography facility in your area contact:

- **The American Cancer Society** 1-800-227-2345
- Or visit the FDA Mammography Facility Database at: http://www.accessdata.fda.gov/scripts/cdrh/cfdocs/cfMQSA/mqsa.cfm

This database is updated from time to time.

Tips for Low Cost Breast Cancer Screening

If you have no insurance or you have limited coverage, these resources may help.

Your Local Hospital. May offer social workers who know about low-cost screenings or special programs in your community. Or, they may be able to help set up a payment plan.

Michigan Breast and Cervical Cancer Control Program.
Provides clinical breast exams, mammograms and diagnostic follow up to women who do not have access to services. **Call 1-800-922-MAMM**

To be eligible, you must be:

- Between 40 and 64 years of age
- Less than 250% of federal poverty income
- Uninsured or underinsured

The American Breast Cancer Foundation (ABCF).
Provides financial assistance to uninsured and underinsured women and men of **<u>all ages</u>** for breast cancer through the Screening Assistance Program. Call their toll-free hotline at 1-877-Key-2-Life (877-539-2543)

Komen Affiliates. Funds breast cancer screening and diagnostic projects for uninsured and underinsured women across the state of Michigan. To find a program near you please call 1-877-GO KOMEN (1-877-465-6636)

Michigan Cancer Genetics Alliance – Directory of Board-Certified Cancer Genetic Specialists in Michigan

Last updated on 11-1-2012

For additional locations visit www.migrc.org

ANN ARBOR

Breast & Ovarian Cancer Risk Evaluation Program University of Michigan Cancer Center
1500 E. Medical Center Dr.
734-764-0107

Cancer Genetics Program Saint Joseph Mercy Hospital
5301 E. Huron River Dr.
734-712-HOPE (4673)

BROWNSTOWN

Oakwood Center for Hematology & Oncology – Downriver
19725 Allen Rd.
734-479-2371

DEARBORN

Oakwood Hospital & Medical Center
18181 Oakwood Blvd.
313-593-8483

DETROIT

Cancer Genetic Counseling Services Karmanos Cancer Institute
4100 John R.
313-576-8748

Henry Ford Health System Cancer Genetics
3031 West Grand Blvd.
313-916-3188

EAST LANSING

Hereditary Cancer Program Michigan State University Genetics
1355 Bogue St.
517-364-5440

FARMINGTON HILLS

Cancer Genetic Counseling Services Karmanos Cancer Institute
31995 Northwestern Hwy
313-576-8748

FLINT

Hurley Medical Center
One Hurley Plaza
810-262-6833

GRAND RAPIDS

Cancer Genetics Program Spectrum Health
100 Michigan St. NE
616-486-6218

The Lacks Cancer Center St. Mary's
200 Jefferson SE
616-685-5600

KALAMAZOO

West Michigan Cancer Center
200 North Park St.
269-373-0126

MARQUETTE

Marquette General Hematology/Oncology
1414 W. Fair Ave
906-225-3922

MIDLAND

MidMichigan Regional Medical Center
4500 Campus Ridge Dr.
989-839-6185

PONTIAC

Cancer Genetics/ Risk Plus Clinic
115 Fulton
248-858-6487

ROCHESTER

Crittenton Hospital Medical Center
1101 W. University Dr.
313-576-8748

ROYAL OAK

Beaumont Cancer Genetics
3577 W. 13 Mile Rd.
248-551-3384

STERLING HEIGHTS

Henry Ford Health System
3500 15 Mild Rd.
313-916-3188

SOUTHFIELD

Academic Internal Medicine
22255 Greenfield Rd.
248-849-3281

TROY

Beaumont Cancer Genetics
4434 Dequindre Rd.
248-551-3388

WEST BLOOMFIELD

Henry Ford Health System
6777 West Maple Rd.
313-916-3188

OTHER AREAS

InformedDNA Telephone Genetic Counseling Services
1-800-975-4819
www.informeddna.com

Michigan Department of Community Health (MDCH)

MDCH can help you find a provider in your area. Call 1-866-852-1247 or email genetics@michigan.gov.

In summary,

- ***Schedule a visit with your health care provider and talk about your breast cancer risk***
- ***Make sure your provider knows that you or your family member had breast cancer at a young age***
- ***Because of this cancer history, you are in a higher-risk group***
- ***Talk about the right screenings for you and what age to begin screening***

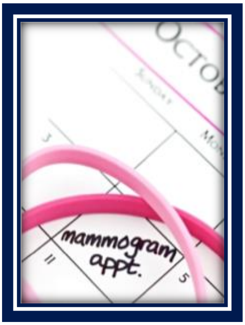

It may be hard to remember and schedule your breast cancer screenings. But, do this as a way to give a gift to yourself. You could also make plans to get your screenings with a family member or friend.

To learn more about breast cancer that runs in families –

- Centers for Disease Control and Prevention:
  1-800-CDC-INFO or www.cdc.gov/cancer/breast
- National Cancer Institute: 1-800-4-CANCER or
  www.cancer.gov/cancertopics/types/breast
- American Cancer Society: 1-800-ACS-2345 or
  www.cancer.org/Cancer/BreastCancer/

Get your mammogram any way you can!
Do it for yourself and your family!

© 2012 Maria Katapodi, PhD, RN, FAAN and Laurel Northouse, PhD, RN, FAAN
University of Michigan School of Nursing
The contents do not necessarily represent official views of the CDC
For questions or copies, contact Maria Katapodi at (734) 647-0178 or mkatapo@umich.edu

Breast Cancer and Your Family: How to Improve Communication and Support

A guide for survivors and their family members

Breast cancer treatments and follow-up care can be stressful for women and their families.

Talking with your relatives about your family's risk for breast cancer also can be very hard to do.

Open family communication can help both survivors and their relatives deal with this stress. It can help you support each other and bring your family closer. It can also help you work as a team to lower your cancer risk.

This brochure has practical and useful tips on

- **how to talk to your relatives about cancer risk**
- **how to find ways to support each other**

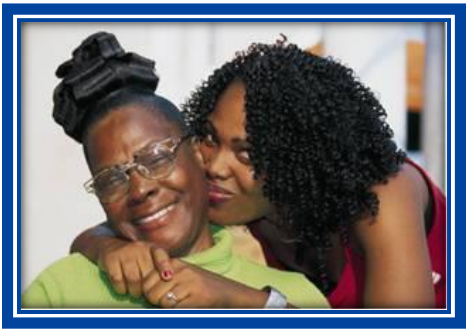

Reach out to each other for support because "A shared burden is half a burden."

Breast cancer and your family

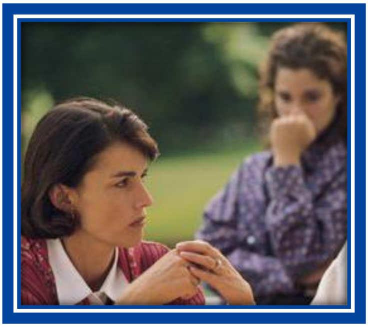

Talking about difficult topics

Breast cancer and cancer risk are hard topics to discuss for almost everyone. **It's normal to feel worried** about your cancer risk. It's also normal to feel upset or uncomfortable when talking about your own journey with breast cancer or your family's history. You may be concerned about how your family members will react or what they will think about trying to lower their own risk.

These topics can make family communication hard

Bringing up the topic of cancer risk in the family **may create tensions and conflict.** Family members might disagree about how breast cancer affects the whole family.

Certain relatives may be very hard to talk to. Some family members may refuse to talk about cancer risk completely. Others may want to blame certain people in the family.

These are the challenges that families like yours may face. But, research has shown that open communication has many benefits for breast cancer survivors and their family members.

Open family communication can help you and your relatives

- cope better with cancer risk
- reduce your stress level
- get support from one another
- view cancer risk as a shared concern
- make better health decisions
- take steps to lower your cancer risk
- make your family stronger and healthier

Tips for better family communication

How to get started

- Think of **who needs to know** about the family's cancer risk
- Write down **the key points** about your family's cancer history that you want to share
- Start with a family member **who is easy to talk to**
- **Pick a time** to talk when you aren't tired
- Choose **a quiet place** without distractions

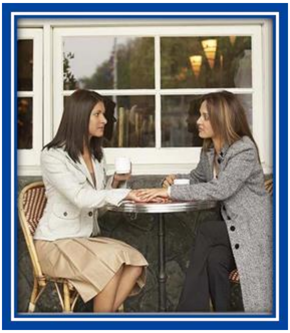

How to be an "active listener"

Most of us think that communicating means talking. But, listening is also very important. Active listening means that you focus your attention on the other person.

- Show you want to listen with a **relaxed face and body**
- Try to understand the **other person's point of view**
- **Ask questions** if you don't understand
- Listen **without interrupting**
- Try hard **not to criticize or argue**. These can keep others from sharing their thoughts and feelings

How to keep the communication open

- Open communication is not a one time thing. It's OK to talk about a big issue like cancer in **small doses.**
- **Be aware** of how your words or actions may affect others.
- Don't push family members to talk. Maybe they are not ready and **they need more time**. Suggest that they check a reliable website (like www.cancer.gov) for information.
- You don't need to talk about cancer all of the time. It's OK to set cancer aside and **talk about other things** in your life.
- **Ask for professional help** if your family is having a hard time.

Tips for better family support

Be sensitive and show affection

It means a lot when you **show you care** with a hug or a pat on the shoulder. It's also helpful if you **just listen** when someone else needs to talk.

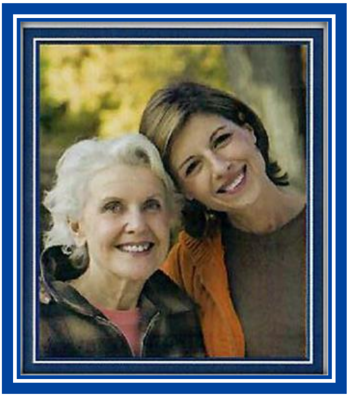

How to work together as a team

You can work as a team to **help each other** lower your breast cancer risk. Here are some ways family members can support each other.

- **Share news** with your relatives about your health. Ask them to do the same. Then, you all can keep your health providers updated with your family's health history.
- **Compare notes** about any breast cancer treatments, prevention steps, and suggested screenings.
- Offer to go along to medical appointments. When family members **attend appointments**, they may learn useful information which helps them support each other.
- Schedule your mammograms and other screenings **together**.
- Help each other **with a car ride or child care** when it's time to get your screenings. **Pass on tips** you get about low cost mammograms in your area.
- Exercise together. **Support each other to follow a healthy lifestyle.** Making healthier choices can lower your cancer risk.
- Plan to have fun family times. This way, you will have more chances to **give and receive support** from each other.

© 2012 Maria Katapodi, PhD, RN, FAAN
Laurel Northouse, PhD, RN, FAAN and Ann Schafenacker, MSN, RN
University of Michigan School of Nursing
in collaboration with the Michigan Department of Community Health
Funded by the Centers for Disease Control and Prevention
The contents do not necessarily represent official views of the CDC
For questions or copies, contact Maria Katapodi at 734 647-0178 or mkatapo@umich.edu

MARIA KATAPODI, Ph.D., R.N., FAAN
Assistant Professor
Robert Wood Johnson Foundation – Nurse Faculty Scholar 2010
400 North Ingalls, Room 2158
Ann Arbor, Michigan 48109-5482
Tel: 734.647.0178
email: mkatapo@umich.edu

Date ________

Name: YBCS
Address
City, MI Zip

Dear _______,

Thank you for taking part in our study. Our goal is to help young breast cancer survivors and their female relatives take the best possible care of their health.

It is important for you to know that having **breast cancer at 45 years of age or younger** is a sign that a woman is in a high-risk group. **High-risk women** may have greater chances of getting another breast cancer than other women. One reason is because cancer that starts at a young age may be caused by changes in genes that are inherited (passed down in the family).

High-risk women may benefit from **talking to a cancer genetic specialist**. Genetic specialists can discuss the chances that your breast cancer has an inherited cause. They can explain genetic testing and how it may help find out if your breast cancer was inherited. They can also suggest things you can do to lower your chances of getting another cancer. Finally, genetic specialists can figure out if other members in your family have higher chances of breast cancer.

Follow-up health care is also important for all breast cancer survivors. Please take a few minutes now to **look over the materials in this envelope.** They have important information about how to locate a cancer genetic specialist and tips for low cost follow-up care that you may need to know about.

Please talk with your health care provider about a **referral to a cancer genetic specialist and follow-up care that is right for you.** For more information, please call 1-800-CDC-INFO or visit www.cdc.gov/cancer/breast.

Thank you once again for taking part in our study. **You will receive a follow-up survey in about 9 months.** Your answers will help us learn more about ways to inform women like you about the importance of cancer screenings and follow-up care.

Sincerely,

MKatapodi

Maria C. Katapodi, PhD, RN, FAAN
Assistant Professor of Nursing
